# Development and preliminary application of a quadruplex real-time PCR assay for differential detection of porcine circovirus 1–4 in Chengdu, China

**DOI:** 10.3389/fvets.2024.1337461

**Published:** 2024-04-30

**Authors:** Yong Mi, Di Huang, Yong Zhuo, Min Li, Jianguo Yue, Hongyu Zhong, Huanhuan Li, Zhijun Zhong, Haifeng Liu, Guangneng Peng, Ling Zhu, Xiaoxiao Zhou, Ziyao Zhou

**Affiliations:** ^1^College of Veterinary Medicine, Sichuan Agricultural University, Chengdu, China; ^2^Chengdu Center for Animal Disease Prevention and Control, Chengdu, China; ^3^Key Laboratory for Animal Disease Resistant Nutrition of the Ministry of Education, Animal Nutrition Institute, Sichuan Agricultural University, Chengdu, China; ^4^Key Laboratory of Agricultural Bioinformatics, Ministry of Education, Sichuan Agricultural University, Chengdu, China

**Keywords:** porcine circovirus, TaqMan multiplex real-time PCR, kit comparison, analysis of epidemic, genetic evolutionary analysis

## Abstract

Porcine circovirus (PCV) typically causes severe immune suppression in pigs, leading to mixed clinical infections with various pathogens that can cause significant harm to the pig industry. PCV has four subgenotypes, with PCV4 being an emerging virus that requires investigation due to its potential for epidemic outbreaks. Therefore, there is a need to develop a method that can detect all four PCV strains simultaneously. In this study, four pairs of specific primers and TaqMan probes were designed based on the conserved sequence of the PCV1–4 ORF2 gene to establish a PCV1–4 TaqMan multiplex real-time quantitative PCR method. The novel method was compared to six commercial testing kits for its efficacy. Then, a total of 595 mixed samples of spleen and lymph node collected from 12 districts in Chengdu from July to December 2021 were tested using the novel method. The results showed that the novel PCV1–4 TaqMan multiplex real-time quantitative PCR detection method has satisfied specificity, sensitivity, and repeatability. The positive rates of PCV1, PCV2, and PCV3 in Chengdu were 2.18%, 31.60%, and 15.29%, respectively, while no positive PCV4 was detected. The mixed infection rate of PCV2 and PCV3 was 5.21%. Our novel method may be as a potential method for PCV1–4 detection. Currently, PCV2 is the main epidemic PCV subtype in Chengdu, while the potential threat of PCV4 should also be considered.

## 1 Introduction

The pig industry is one of the pillar industries in the world, playing a crucial role in ensuring people's livelihoods and promoting economic growth (1, 2). However, the industry has been facing challenges due to the prevalence of porcine circovirus (PCV), which is often referred to as the “invisible killer,” that cause recessive and mixed infections of multiple pathogens without typical clinical symptoms (3). PCV can be subdivided into four genotypes, namely porcine circovirus type 1–4 (PCV1–4), based on differences in nucleotide homology. Among these, PCV4 is a novel circovirus that was first discovered in 2019 and is currently endemic only in Asia, mainly in China (4–7).

The complete genome size of PCV is ~1.7–2.0 kb (8). The ORF2 is located on the negative strand of the virus and encodes the capsid protein (Cap), which is one of the most crucial antigenic sites among all four subtypes of PCV (9, 10). As the outermost structural component of the virus, the Cap protein directly interacts with the host immune system, resulting in substantial immunological pressure (11–13). Consequently, it undergoes a high rate of amino acid variation in order to evade immune responses (14). When comparing the amino acid sequences of Cap proteins from PCV subtypes 1–4, the homology ranges from 24.5 to 64% (7, 9). Since the significant antigenic response and the lower genetic relatedness of ORF2, it can be used for detecting and distinguishing different subtypes of PCV (15–17).

Currently, commercial diagnostic kits for PCV primarily focus on detecting PCV2 (18), which may overlook the detection of other subtypes (19). Therefore, there is a need to develop a method that can detect all four PCV strains simultaneously. Additionally, it is unclear about the specific infection situation in Chengdu, which is one of the most renowned pig farming regions in Southwest China (20). This study established a TaqMan multiplex real-time quantitative PCR (qPCR) detection method for PCV1–4, evaluated its amplification efficiency and accuracy, and compared it with a commercial detection kit. Furthermore, the method was preliminarily applied in Chengdu.

## 2 Materials and methods

### 2.1 Primer probe design

ORF2 gene sequences of PCV1–4 from different countries published in NCBI GeneBank were selected for sequence alignment using SnapGene software (GSL Biotech; available at snapgene.com). The highly conserved region in ORF2 gene was selected to design primer probes using OLIGO 7 primer analysis software (21). The designed primers were compared with NCBI for Blast to verify the specificity. Primers and probes were synthesized by Qink Bio (Chongqing) Co., Ltd., listed in Table 1.

**Table 1:** Primers and probe.

**Primer**	**Sequence (5^′^-3^′^)**	**PCR length**
PCV1-F	CAACTTGGCCTATGACCCCTA	127 bp
PCV1-R	TGGGTGGAACCAATCAATTGT	
PCV1-Probe	CY5-CCTCCCGCCACACCATAAGGCAGC-BHQ-2	
PCV2-F	CACCGTTACCGCTGGAGAAG	93 bp
PCV2-R	CGTTCTGACTGTGGTTTTCTTG	
PCV2-Probe	FAM-ATGGCATCTTCAACACCCGCCTCT-BHQ-2	
PCV3-F	AGTGCTCCCCATTGAACGGTG	136 bp
PCV3-R	AACACAGCCGTTACTTCACC	
PCV3-Probe	HEX-ACCCACCCCATGGCTCAACACA-BHQ-1	
PCV4-F	TCTCACTGTCCACACCTGCAC	126 bp
PCV4-R	CTCCACTTCCAGCCTAACAAC	
PCV4-Probe	ROX-CCTGGTCCGCCATGCTGATCCAC-BHQ-2	

### 2.2 Primer probe design plasmids synthesis

A set of plasmids containing the complete genomes of PCV1–4 were designed based on the reference sequences for PCV1–4 types available in GenBank (accession numbers: EF533941, AF201310, MG778698, MT311852, respectively). These plasmids were synthesized (Qink Bio, Chongqing Co., Ltd.) at a concentration of 100 ng/μl, named pUC57-PCV1-Complete, pUC57-PCV2-Complete, pUC57-PCV3-Complete, and pUC57-PCV4-Complete, respectively.

The pClone007 Simple Vector Kit was used to perform T-A ligation. Trelief TM 5α competent cells were used for transformation immediately following the instructions. The transformed cells were incubated in LB medium containing 50 μg/ml Ampicillin at 37°C for 12 h. Monoclonal colonies were then inoculated in LB liquid medium and shaken overnight.

### 2.3 Optimization of TaqMan multiplex real-time fluorescence quantitative PCR amplification reaction conditions

The TaqMan multiplex real-time fluorescence quantitative PCR amplification was performed using the specific primers and probes designed in this experiment, with the recombinant plasmid DNA as the template. The amplification was performed with the reaction conditions: initial denaturation at 95°C for 5 min, followed by 30 cycles of denaturation at 95°C for 10 s and annealing/extension at 60°C for 50 s. The original reaction system shown in Table S1.

A matrix optimization was performed for the parameters, including the concentrations of primers (0.05, 0.1, 0.15, 0.2, 0.25, 0.3, 0.35, 0.4, 0.45, and 0.5 μM), probe (0.01, 0.05, 0.1, 0.15, 0.2, 0.25, 0.3, 0.35 μM), annealing temperatures (65, 64, 63, 62, 61, 60, 59, and 58°C) and cycle number (30, 35, and 40). The smallest Ct value was used for determining the excellent reaction conditions. When the Ct values were the same, the higher annealing temperature was selected.

### 2.4 Verification of the novel method

The positive standard plasmids were diluted to 1 × 10^3^-1 × 10^7^ copies/μl. 0.5 μl of each plasmid were selected as the templates for reaction system. The negative control template was 0.5 μl of dd H_2_O. Multiple quantitative PCR amplification was performed according to the optimized reaction conditions. The amplification results were analyzed using Bio-Rad CFX Maestro, a quantitative PCR data analysis software, to establish standard curves. The standard plasmids were further diluted to 1 × 10^4^-1 × 10^0^ copies/μl, that were selected as the template for performing the sensitivity test.

In order to better simulate clinical examination and study its specificity and repeatability, we randomly diluted different concentrations of the four viral plasmids, detected their plasmid concentrations by A260/A280 (Qubit 4 Fluorometer, Thermo Fisher Scientific). DNA or reversed cDNA of PRV, ASFV, PPV, and PRRSV, PoRV, PEDV and PK-15 cell line and blank DH5α competent cells were selected as templates for verify the specificity of TaqMan multiplex real-time PCR method. The TaqMan multiple real-time PCR amplification was performed for three repetitions.

The above positive standards were collected and tested three times at every week interval under the same reaction conditions, which was used as the repeated test between groups. The intragroup and intergroup coefficients of variation were calculated to verify the reproducibility of the established TaqMan multiplex real-time PCR method.

### 2.5 Comparison of our novel method to six commercial kits

For further confirming the detection accuracy of our method, six commercial TaqMan real-time fluorescence quantitative PCR detection kits for PCV were purchased from three different manufacturers, named A1, A2, B1, B2, C1, and C2 (specific parameters for each kit listed in Table S2). The plasmids containing complete genomes of PCV1–4 were random diluted (Table S3) and then detected by our method and all the commercial kits. Please note the specific parameters may be various according to the manufacturer manual. Thirty mixed samples of spleen and lymph node collected from Chengdu humane slaughterhouse were also detected as blind samples.

### 2.6 Preliminary application of our method for Chengdu

From July to December 2021, a total of 595 spleen and lymph node mixed samples from humane slaughterhouse were collected from 15 districts in Chengdu. The positive control used the plasmids containing the complete genomes of PCV1–4 described previously. The preliminary application of our method for Chengdu was performed for the detection of PCV1–4.

## 3 Results

### 3.1 Preliminary TaqMan multiplex real-time fluorescence quantitative PCR

**Figure 1:**
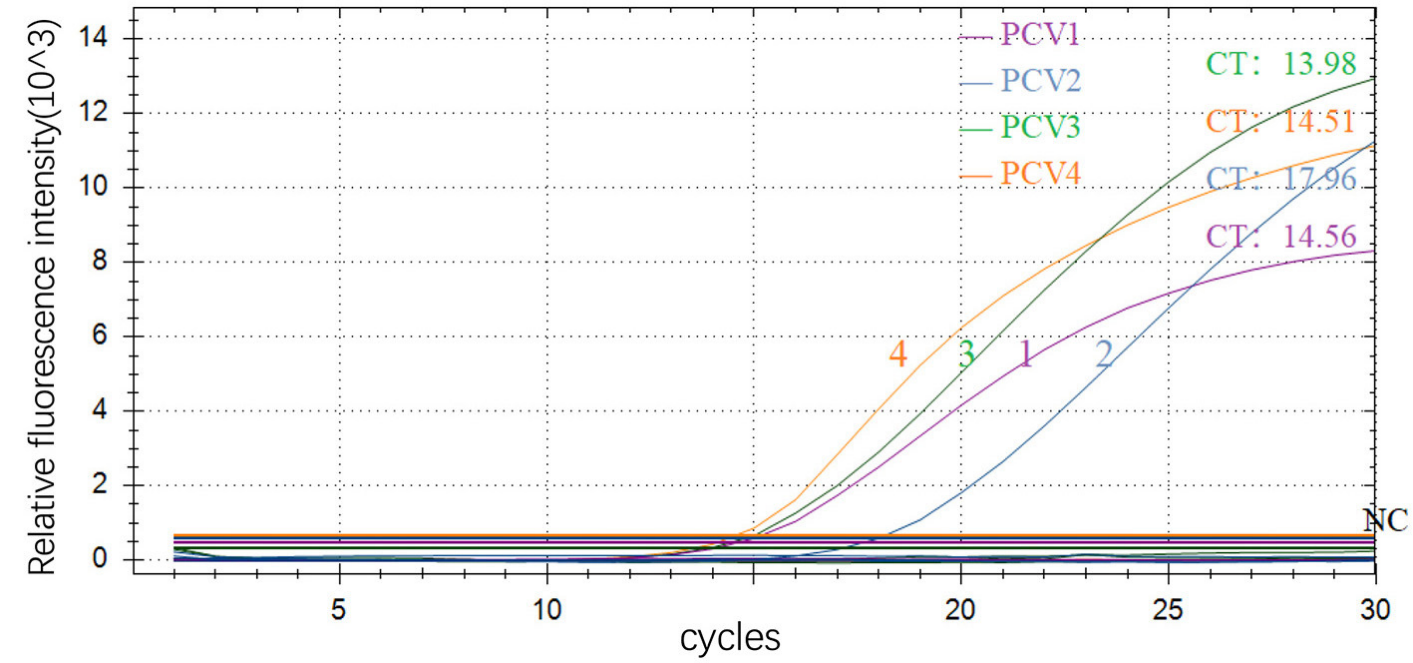
Preliminary amplification results of multiple real-time fluorescence quantitative PCR 1–4, PCV1, PCV2, PCV3 and PCV4; NC: negative control.

The amplification results showed typical amplification curves for all four positive plasmids, while not for negative control, indicating that the preliminary designed primers and probes have good potential for further optimization (Figure 1).

For optimization of reaction conditions, at 59–61°C under the same template concentration, the amplification efficiency and fluorescence intensity were consistent, with little change of Ct value. Since higher annealing temperature may reduce non-specific amplification, the annealing temperature was finally chosen to be 61°C. For the final primer concentration optimization, PCV1, PCV3, and PCV4 required a primer concentration of 0.05 μmol/ml, while PCV2 required a concentration of 0.15 μmol/ml. The probe concentration was set to 0.01 μmol/ml for all targets (Table 2).

**Table 2:** The optimized multiplex qPCR reaction system.

**component**	**Amount (**μ**l)**			
	**PCV1**	**PCV2**	**PCV3**	**PCV4**
2 × T5 fast qPCR mix	10			
10 μM/ml primer-up	0.1	0.3	0.1	0.1
10 μM/ml primer-down	0.1	0.3	0.1	0.1
10 μM/ml probe	0.02	0.02	0.02	0.02
Template	0.5			
ddH_2_O	8.22			
Total	20			

**Figure 2:**
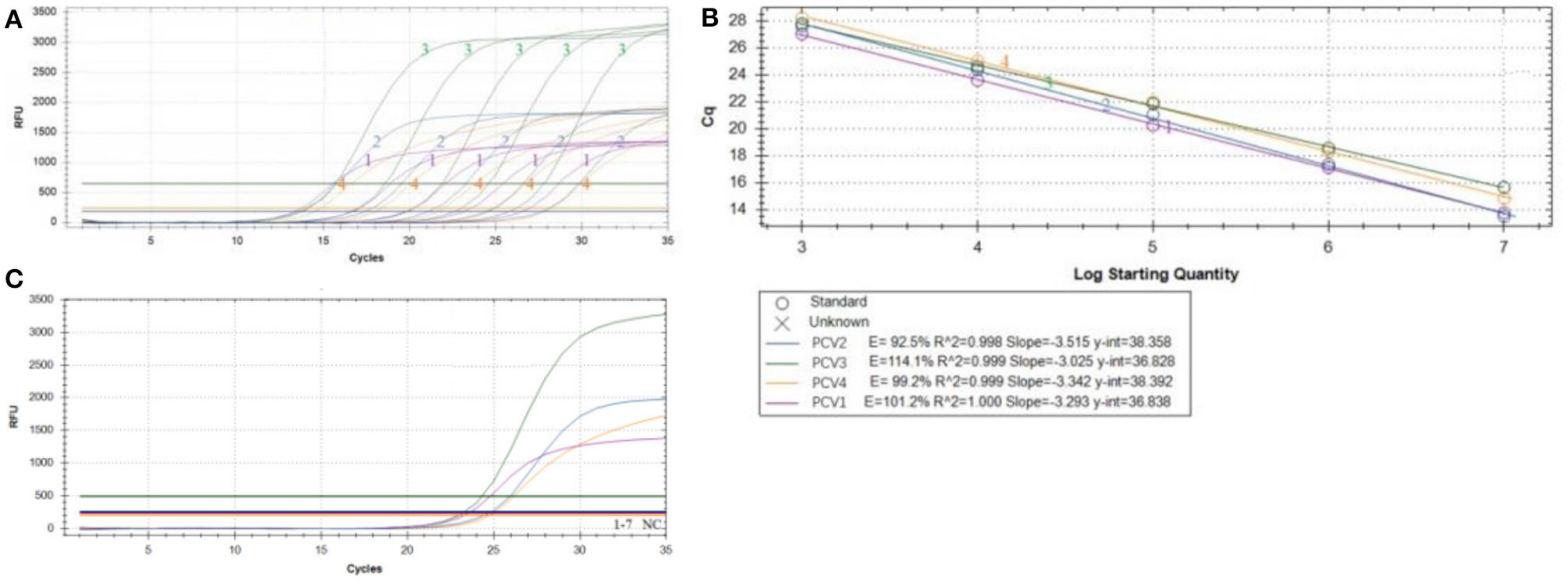
Multiplex qPCR amplification curve. The amplification curve **(A)** and standard curve **(B)** of TaqMan multiplex real-time PCR using positive standard plasmid with concentration gradient of 1 × 10^3^-1 × 10^7^copies/μl; **(C)** Specificity of multiplex qPCR amplification of PCV1–4 PRV, ASFV, PPV, PRRSV, PoRV, PEDV and PK-15 cell, please note the original Plasmid concentration of PCV1–4 were: PCV1 5.86 × 10^1^ copies/μl, PCV2 9.69 × 10^2^ copies/μl, PCV3 6.54 × 10^1^ copies/μl, PCV4 5.11 × 10^1^ copies/μl, respectively.

After optimization, the amplification curve (Figure 2A) and standard curve (Figure 2B) of TaqMan multiplex real-time PCR showed a good linear relationship between Ct value (*y*) and positive standard copy number (*x*) in the gradient concentration range of 10^7^-10^3^ copies/μl. The standard curves corresponding to the species target genes were as follows: PCV1: *y* = −3.293*x* + 36.838, *R*^2^ = 1.000, amplification efficiency (*E*) = 101.2%; PCV2: *Y* = −3.515*x* + 38.358, *R*^2^ = 0.998, amplification efficiency (*E*) = 92.5%; PCV3: *y* = −3.025*x* + 36.828, *R*^2^ = 0.999, amplification efficiency (*E*) = 114.1%; PCV4: *y* = −3.342*x* + 38.392, *R*^2^ = 0.999, amplification efficiency (*E*) = 99.2%.

### 3.2 Sensitivity, specificity and repeatability of our novel detect method

The TaqMan multiplex real-time PCR method showed a good minimum copy number detection limit for each of the PCV targets: PCV1 (5.86 × 10^1^ copies/μl), PCV2 (9.69 × 10^2^ copies/μl), PCV3 (6.54 × 10^1^ copies/μl), and PCV4 (5.11 × 10^1^ copies/μl). Specificity testing demonstrated that only the PCV plasmid yielded typical amplification curves, while other pathogens and cells (including PRV, ASFV, PPV, PRRSV, PoRV, PEDV, and PK-15 cells) did not show amplification curves, indicating the method's good specificity (Figure 2C). For repeatability, the intragroup coefficient of variation ranged from 0.33 to 2.48%, and the intergroup coefficient of variation ranged from 0.53 to 3.59% (Table 3), all of which were < 5%, indicating that the established method exhibited good repeatability.

**Table 3:** Repeatability test results of multiplex qPCR.

**Amplification template**	**Copies**	**Intragroup variation**		**Intergroup variation**	
		**Ct (** $\bar{X}\pm s$ **)**	**CV/%**	**Ct (** $\bar{X}\pm s$ **)**	**CV/%**
PCV1	5.86 × 10^6^	17.05 ± 0.25	1.47%	17.28 ± 0.45	2.60%
	5.86 × 10^5^	20.24 ± 0.36	1.78%	20.06 ± 0.72	3.59%
	5.86 × 10^4^	23.56 ± 0.47	1.99%	23.71 ± 0.27	1.14%
PCV2	9.69 × 10^6^	17.57 ± 0.19	1.08%	17.84 ± 0.22	1.23%
	9.69 × 10^5^	21.09 ± 0.22	1.04%	20.99 ± 0.51	2.43%
	9.69 × 10^4^	24.78 ± 0.26	1.05%	24.35 ± 0.13	0.53%
PCV3	6.54 × 10^6^	18.15 ± 0.45	2.48%	18.44 ± 0.19	1.03%
	6.54 × 10^5^	21.67 ± 0.57	2.63%	21.55 ± 0.24	1.11%
	6.54 × 10^4^	24.99 ± 0.32	1.28%	25.25 ± 0.68	2.69%
PCV4	5.11 × 10^6^	18.16 ± 0.06	0.33%	18.63 ± 0.33	1.77%
	5.11 × 10^5^	21.66 ± 0.17	0.78%	21.52 ± 0.19	0.88%
	5.11 × 10^4^	25.25 ± 0.54	2.14%	24.77 ± 0.49	1.98%

### 3.3 Comparison of our novel method to six commercial kits

The comparison between our novel method and six different commercial test kits revealed that the lowest detectable copy number ranged from 5 × 10^1^ copies/μl to 5 × 10^3^ copies/μl in different genotypes. In most cases, our novel method detected similar or fewer copies than the commercial test kits (Table S4). The coefficient of variation for all seven methods ranged from 0.52 to 2.86%, indicating good reproducibility and suggesting that both the novel method and the commercial test kits are reliable (Table S5). Using 30 clinical mixed spleen and lymph node samples, we found that the novel method and test kits provided similar results to the standard plasmids. Of the 30 tissue samples, the agreement rates between our novel method and the test kits ranged from 73.33 to 96.67%, with significant correlation coefficients of 0.877–0.977 (Table S6). However, no samples were detected with PCV4. Overall, our novel method performed comparably or even better than the commercial test kits, indicating that it could be a viable alternative for PCV detection in clinical samples.

### 3.4 Clinical detection using the TaqMan multiplex real-time fluorescence quantitative PCR

Using the TaqMan multiplex real-time fluorescence quantitative PCR method established in this study, we tested a total of 595 spleen and lymph node mixed samples from dead pigs sent to harmless treatment plants from slaughterhouse in 15 districts in Chengdu city from July to December 2021 for PCV. The total number of PCV-positive samples was 292, resulting in a positive rate of 49.07%, with a mixed infection positive rate of 6.39% (Table 4). The infection was still dominated by PCV2 and PCV3 mixed infection, accounting for approximately 81% of the total mixed infections.

**Table 4:** Results of clinical detected positive samples from Chengdu, July to December 2021.

**PCV1**		**PCV2**		**PCV3**		**PCV4**		**Total**	
**Positive sample**	**Positive rate**	**Positive sample**	**Positive rate**	**Positive sample**	**Positive rate**	**Positive sample**	**Positive rate**	**Positive sample**	**Positive rate**
13	2.18%	188	31.60%	91	15.29%	0	0.00%	292	49.07%

## 4 Discussion

PCV1 was first identified as a PK15 porcine kidney cell line contaminant in 1974. PCV2 and PCV3 were subsequently isolated and identified in 1991 and 2016, respectively (9, 22). Both types of viruses are highly infectious to pigs and often cause PMWS, PDNS, PRDC, and PNP in clinical practice. PCV4 was first discovered in Hunan Province in 2019 (9, 23, 24) and has been detected in several other provinces in China as well as in South Korea (25, 26).

In this study, a convenient PCV1–4 TaqMan multiplex real-time PCR detection method was established based on the same principle as ordinary PCR. However, this multiplex PCR method adds multiple primer pairs to the reaction system to obtain multiple target genes through specific amplification of different templates or different regions of the same template. Real-time quantitative PCR methods are mainly divided into SYBR dye method and TaqMan probe method (27). Compared with ordinary PCR and SYBR dye method, TaqMan probe method avoids false positives caused by primer dimer, does not require a dissolution curve, and has high specificity, making it possible to detect multiple pathogens at the same time (28, 29).

Specificity and sensitivity are two basic characteristics of PCR diagnostic reagents and also two important indexes that directly determine the performance of the reagents (30). Factors affecting these characteristics in PCR reactions include primer design, primer concentration, annealing temperature, substrate concentration, elongation time (31–33). Generally, excessively high primer concentration leads to increased non-specific amplification products, while excessively low primer concentration does not meet the needs of the exponential amplification period, both of which affect amplification efficiency. Similarly, excessively high or low probe concentration affects the accuracy of experimental results (34). Therefore, this experiment optimized primer and probe concentrations using the matrix method to excessive primers and probes. In this study, amplification efficiency, fluorescence signal intensity, and non-specific amplification of primer probes with different concentrations were analyzed to determine the two groups of primer probe concentrations with the optimal ratio. Increasing the annealing temperature can reduce non-specific binding between the primer and template, make the DNA double strands unchain more thoroughly, and improve the amplification efficiency and specificity of PCR reaction, but reducing the annealing temperature can improve the amplification yield. The temperature gradient of 58–65°C was set in this experiment, and the final annealing temperature was chosen as 61°C. The number of PCR cycles mainly depends on the concentration of template DNA, which is generally 25–35 times. In this experiment, the number of cycles was optimized under the above optimized reaction conditions, and 35 cycles were chosen.

Current studies on PCV real-time PCR methods include several studies that have established minimum detection limits for PCV1, PCV2, PCV3, and PCV4. For instance, Li et al. (35) established minimum detection limits of 1 × 10^1^ copies/μl and 1 × 10^2^ copies/μl for PCV1 and PCV2, respectively, using a dual real-time PCR method. Moreover, Li et al. (36) established the lowest detection limits of 2.9 × 10^0^ copies/μl and 2.25 × 10^1^ copies/μl for PCV2 and PCV3, respectively, using a dual real-time fluorescence quantification method. Chen et al. (4) established a quadruplex real-time PCR method for PCV1–4, with a lowest copy number detection limit of 2.8 × 10^1^ copies/μl for each genotype. In this study, the lowest copy number detection limit of PCV2 was 9.69 × 10^2^ copies/μl, which was slightly higher than that of previous studies. Nonetheless, this method still meets the needs of clinical detection.

By utilizing the TaqMan multiplex real-time fluorescence quantitative PCR method developed in our study, a total of 292 samples tested positive for PCV, resulting in a positivity rate of 49.07%. Among these samples, there were 38 cases of mixed PCV infections, with a mixed infection positivity rate of 6.39%. Notably, PCV2 and PCV3 co-infections were prevalent among the mixed infections, accounting for 31 out of the 38 cases. When compared to findings from other researchers, our study showed consistent positivity rates for both PCV single infections and mixed infections. However, the regional distribution of PCV yielded intriguing results, revealing that PCV2 and PCV3 were detected in all districts and cities, except for the five urban areas (Wuhou, Qingyang, Jinjiang, Chenghua, and Jinniu) where pig farming activities were absent. Furthermore, these areas exhibited comparatively high positivity rates. Although, the potential impact of varying viral loads at different tissue locations may result the variation of detection rates, our detect in Chengdu is relatively similar with other regions of China (37–39). This suggests that PCV remains a hidden threat to pig populations in the Chengdu region. Although pigs in Chengdu have not been infected with PCV4, protective measures should still be taken. Farmers should continue to strengthen the management of feeding and the feeding environment to reduce the cross-infection of viruses.

In conclusion, our novel method may provide for PCV1–4 detection. Currently, PCV2 is the main popular in Chengdu, while the potential threat of PCV4 should also be considered.

## Data availability statement

Publicly available datasets were analyzed in this study, with the NCBI GenBank accession numbers: EF533941, AF201310, MG778698, MT311852.

## Author contributions

YM: Writing – original draft, Investigation, Writing – review & editing. DH: Writing – review & editing, Conceptualization, Investigation, Methodology, Writing – original draft. YZ: Writing – original draft, Investigation, Validation, Writing – review & editing. ML: Writing – review & editing, Methodology, Resources. JY: Resources, Writing – review & editing. HZ: Writing – review & editing, Investigation. HLi: Writing – original draft. ZZhon: Writing – review & editing, Methodology. HLiu: Writing – original draft, Data curation. GP: Writing – review & editing, Resources. LZ: Resources, Writing – review & editing. XZ: Writing – review & editing, Resources, Supervision. ZZhou: Writing – review & editing, Conceptualization, Supervision, Writing – original draft.
