# Supplementary material for: Development and preliminary application of a quadruplex real-time PCR assay for differential detection of porcine circovirus 1–4 in Chengdu, China
Source: Front Vet Sci. 2024 Apr 30;11:1337461. doi: 10.3389/fvets.2024.1337461 (PMC11091263; doi:10.3389/fvets.2024.1337461)
Supplement: Supplementary file 1 [file Data_Sheet_1.PDF]

Table S1. Multiplex qPCR reaction system

component	amount
2 × T5 Fast qPCR Mix	10 μL
10 μM primer-F	0.8 μL
10 μM primer-R	0.8 μL
10 μM probe	0.4 μL
Recombinant plasmid DNA	0.5 μL of each plasmid
ddH_2O	6 μL
Sum	20 μL

Table S2. The parameters of commercial test kit

kits	test	cycles	Reaction system volume/μL	Template volume/μL	result interpretation
A1	PCV1<br>PCV2	40	20	2	Ct≤ 30, positive; 30< Ct< 35, suspicious; Ct≥ 35, negtive
A2	PCV2<br>PCV3	40	20	2	Ct⩽ 30, positive; 30< Ct< 35, suspicious; Ct⩾ 35, negtive
B1	PCV2	40	25	5	Ct⩽ 30, positive; 30< Ct< 35, suspicious; Ct⩾ 35, negtive
B2	PCV3	40	25	5	Ct⩽ 30, positive; 30< Ct< 35, suspicious; Ct⩾ 35, negtive
C1	PCV2<br>PCV3	45	25	5	Ct⩽ 35, positive; 35< Ct< 40, suspicious; Ct⩾ 40, negtive
C2	PCV4	45	25	5	Ct⩽ 35, positive; 35< Ct< 40, suspicious; Ct⩾ 40, negtive

Table S3. Random dilution of positive plasmid

plasmid	ng/μL	A260/A280	copies/μL
PCV1	81.4	1.99	5.86×10^{11}
PCV2	99.7	1.90	9.69×10^{11}
PCV3	97.4	1.95	6.54×10^{11}
PCV4	70.6	1.96	5.11×10^{11}

Table S4. Sensitivity of comparasion of methods

plasmid	PCV1		PCV2					PCV3				PCV4	
kits	TS	A1	TS	A1	A2	B1	C1	TS	A2	B2	C1	TS	C2
detection limit (copies/μL)	50	500	500	500	500	500	50	50	5000	500	5	50	5

TS: this study.

Table S5. The sensitivity test results of our method and each kit

plasmid	methods	template concentration (copies/μL)			Ct(X ± s)	CV/%
		5×10^3				
		Repeat 1	Repeat 2	Repeat 3		
PCV1	TS	26.97	27.35	27.06	27.13 ± 0.20	0.73%
	A1	24.69	25.06	25.44	25.06 ± 0.38	1.50%
PCV2	TS	28.30	27.93	27.91	28.05 ± 0.22	0.78%
	A1	29.52	29.85	30.00	29.79 ± 0.25	0.82%
	A2	29.47	28.56	28.97	29.00 ± 0.46	1.57%
	B1	30.35	30.20	31.09	30.55 ± 0.48	1.56%
	C1	34.20	34.66	35.02	34.6 ± 0.41	1.19%
PCV3	TS	27.82	28.14	27.90	27.95 ± 0.17	0.60%
	A2	32.77	33.68	32.05	32.83 ± 0.82	2.49%
	B2	29.36	30.58	31.04	30.33 ± 0.87	2.86%
	C1	27.55	28.07	27.11	27.58 ± 0.48	1.74%
PCV4	TS	28.62	28.35	28.59	28.52 ± 0.15	0.52%
	C2	26.48	27.00	27.53	27.00 ± 0.53	1.94%

TS: this study.

Table S6. Comparison results of coincidence rate and correlation coefficient between our method and commercial kits in clinical samples

methods	Coincidence rate, correlation coefficient			
	TS PCV1	TS PCV2	TS PCV3	TS PCV4
A1-PCV1	96.67% 0.879	-	-	-
A1-PCV2	-	90.00% 0.949	-	-
A2-PCV2	-	90.00% 0.958	-	-
B1-PCV2	-	90.00% 0.977	-	-
C1-PCV2	-	86.67% 0.877	-	-
A2-PCV3	-	-	93.33% 0.938	-
B2-PCV3	-	-	93.33% 0.911	-
C1-PCV3	-	-	73.33% 0.910	-
C2-PCV4	-	-	-	100.00% -

TS: this study.
